# Diabetes, pre-diabetes and their risk factors in Malta: a study profile of national cross-sectional prevalence study

**DOI:** 10.1017/gheg.2016.18

**Published:** 2016-11-23

**Authors:** S. Cuschieri, J. Vassallo, N. Calleja, N. Pace, J. Mamo

**Affiliations:** 1 Department of Anatomy, Faculty of Medicine and Surgery, University of Malta, Msida, Malta; 2 Department of Medicine, Faculty of Medicine and Surgery, University of Malta, Msida, Malta; 3 Department of Public Health, Faculty of Medicine and Surgery, University of Malta, Msida, Malta

**Keywords:** Cross-sectional studies, diabetes mellitus, evidence-based medicine, health care survey, Malta

## Abstract

**Background:**

Type 2 diabetes mellitus constitutes a global epidemic and a major burden on health care systems across the world. Prevention of this disease is essential, and the development of effective prevention strategies requires validated information on the disease burden and the risk factors. Embarking on a nationally representative cross-sectional study is challenging and costly. Few countries undertake this process regularly, if at all.

**Method:**

This paper sets out the evidence-based protocol of a recent cross-sectional study that was conducted in Malta. Data collection took place from November 2014 to January 2016.

**Results:**

This study presents up-to-date national data on diabetes and its risk factors (such as obesity, smoking, physical activity and alcohol intake) that will soon be publicly available.

**Conclusion:**

This protocol was compiled so that the study can be replicated in other countries. The protocol contains step-by-step descriptions of the study design, including details on the population sampling, the permissions required and the validated measurement tools used.

## Introduction

Type 2 diabetes mellitus (T2DM) is a global epidemic, and the International Diabetes Federation (IDF) declared it a ‘global emergency’ in 2015. The estimated global prevalence of T2DM in 2015 was 8.8% and this is predicted to rise to 10.4% by 2040 [1]. Across Europe, ageing, inadequate physical activity levels, high calorie diets and rising levels of obesity continue to contribute to the disease, which has a substantial impact on individuals' quality of life [2]. Another established risk factor for diabetes is pre-diabetes, where an individual's blood glucose level is above the normal level but not high enough for a diagnosis of diabetes [3]. Those with pre-diabetes are at an increased risk of developing diabetes, with a 5-year conversion rate from 10 to 23% depending on the diagnostic criteria used [4]. Therefore, a greater understanding of pre-diabetes is essential for the prevention of diabetes [3]. In addition, identifying individuals with pre-diabetes at an early stage can have a positive impact on health outcomes [5].

In order for countries to be in a position to manage the increasing burden of diabetes and to be able to develop evidence-based prevention strategies, regular evaluations of the prevalence of diabetes, its risk factors and its impact need to be carried out at the national level [6]. The IDF has strongly recommended that individual countries carry out research (including prevalence studies) in order to obtain an accurate and up-to-date picture of the local diabetes situation [1].

Malta is an archipelago of islands in the Mediterranean Sea, at the crossroads between Europe and Africa, and it has been known to have a high prevalence of T2DM since the 18th century [7]. It is a small archipelago of islands (<316 km^2^) with an accessible population (<500 000 people), which makes it a relatively easy location in which to carry out nationally representative studies on diabetes and other common non-communicable diseases. The increase in the prevalence of diabetes in the country has been attributed to the shift in its population from a Mediterranean lifestyle to a more Westernized lifestyle [8].

Prevalence studies are not conducted regularly in Malta. Effectively, this study protocol is the *only* national cross-sectional study on diabetes and its risk factors to be conducted in the past 35 years [9]. Considering the extensive changes in lifestyles, culture and demographics of the Maltese population over the recent decades and considering the improvements in genetic technology, conducting national surveys of non-communicable diseases such as diabetes that utilize the advanced genetic technology is imperative in order to gather up-to-data data [8]. These national studies should also identify the most important current risk factors for the disease in question.

The study based on this protocol was supported by Malta's Minister for Health who highlighted that it would provide key evidence for the development of prevention strategies and it would provide information to inform future research [10]. As a result of the vital nature of the data collected in the study, a campaign (with stringent ethical criteria) to raise funds from academic and private sources was launched, thereby avoiding the need to rely on the extremely limited state funding.

The study was conducted by the University of Malta. It was entitled ‘SAHHTEK’ (Your Health) – The University of Malta Health and Wellbeing Study' and it took place from November 2014 to January 2016 with encouragement and in-kind support from the government of Malta. The aim of the study was to obtain up-to-date information on the prevalence of T2DM in Malta, to investigate the pre-diabetic population and to shed light on the important links between pre-diabetes and diabetes and its determinants [11]. This paper presents the study protocol and the lessons learnt from the study in order to aid other countries that wish to undertake a similar survey.

### Definition of cases

The participants that had been previously diagnosed with diabetes and were on medication were categorized as having diabetes irrespective of their fasting blood glucose (FBG) levels. Participants who had not previously been diagnosed but who had an FBG level of ≥7 mmol/L were categorized as having newly diagnosed diabetes. This was in line with the standard recommendations to use an FBG level of ≥7 mmol/L for the estimation of the prevalence of diabetes (with no repeat testing if a diagnosis of diabetes is established) [12–14]. Participants with an FBG level of 5.6–6.9 mmol/L who had not previously been diagnosed with diabetes were categorized as having impaired fasting glucose (IFG) [11]. The FBG cut-off point of 5.6 mmol/L has been proven to be the optimal value for the prediction of future diabetes [15]. Participants with a FBG level of <5.59 mmol/L were assumed to have normal carbohydrate metabolism [12].

A 2-h oral glucose tolerance test (OGTT) was undertaken by those who had IFG in order to assess whether the participants had impaired glucose tolerance (IGT) or diabetes (despite not having FBG levels of ≥7 mmol/L). This is in line with the fact that approximately 31% of individuals with diabetes are not picked up by the FBG criteria [13, 16]. In line with the OGTT cut-off criteria of the World Health Organization (WHO), those with a 2-h blood glucose level of 7.8–11.0 mmol/L were categorized as having IGT, whereas those with a level of ≥11.1 mmol/L were categorized as having diabetes [13].

### Sampling methodology

The sample was randomly selected from the official population register in order to be representative of the Maltese resident population (including foreign-born residents) who had lived in Malta at least 6 months. The sample was selected using a stratification strategy which depended on age (between 18 and 70), gender and location. Based on the American Diabetes Association (ADA) estimate of the prevalence of pre-diabetes (25%), the prevalence of pre-diabetes in Malta was estimated to be 25% [17]. Since a primary aim of the study was to identify the subpopulation of individuals with pre-diabetes, it was assumed that the size of the sample would have to be approximately four times the number of individuals with pre-diabetes required. Should the prevalence of individuals with pre-diabetes in Malta be higher than 25%, it would be to the study's advantage. Based on local health examination studies, a response rate of around 50% was expected [18]. Therefore, the size of the sample had to be eight times the required number of individuals with pre-diabetes. In addition, as one of the aims of the study was to explore the associations between a large numbers of factors, for the purpose of sample size estimation, it was assumed that the worst-case scenario was a 50% response rate.

Using the PiFace^®^ software with a maximum confidence interval of ±5%, it was estimated that a sample of 384 participants with pre-diabetes should be recruited [17, 19]. Therefore, a minimum sample of 3072 had to be selected from the population. In order to allow for a lower response rate compared to that in Malta's 2010 European Health Examination Survey, and for deaths and emigration during the fieldwork period, a sample of 4000 was chosen [18]. This amounted to approximately 1% of the adult population of Malta. In fact, the demographics of the study population were similar to the representative data obtained from the latest Maltese demographic report issued by Malta's National Statistics Office, as seen in Table 1 that demonstrates the sample population in relation to the total population of Malta by age groups [20].

**Table 1.** Size of the study sample compared to the size of the national population in 2013

Age group	Total population^a^	Total sample (approximately 1% of population)
18–24	40 564	504
25–34	62 180	747
35–44	56 575	725
45–54	55 113	749
55–64	59 268	804
65–70	33 480	471

Age group	Male population^a^	Male sample (approximately 1% of population)
18–24	21 045	260
25–34	32 359	391
35–44	29 143	377
45–54	27 728	366
55–64	29 585	408
65–70	16 136	239

Age group	Female population^a^	Female sample (approximately 1% of population)
18–24	19 519	244
25–34	29 821	356
35–44	27 432	348
45–54	27 385	383
55–64	29 683	396
65–70	17 344	232

a Demographic data from Malta's National Statistics Office.

The selection of a sample drawn from the population register allowed nationally representative data to be collected that means that conclusions can be drawn for the whole population, and it also means that in-depth analyses of subgroups (such as the diabetic and pre-diabetic subgroups) can be carried out. As single-stage random sampling was used, there was no clustering effect. Pregnant female participants were excluded from the survey

### Permissions

Both the Research Ethics Committee of the Faculty of Medicine and Surgery at the University of Malta and Malta's Information and Data Protection Commissioner gave their permission for the study to be run. The Ministry for Energy & Health, through the Parliamentary Secretary and the Department of Primary Care, provided support in the form of the use of local government health clinics and the use of the Biochemistry Laboratory of the public Mater Dei Hospital.

### Recruitment of fieldworkers

Interviewers and health assessors (who were trained phlebotomists who collected the blood samples as well as carrying out the health examinations) were recruited using adverts in local newspapers. The ability to communicate in both Maltese and English was considered to be essential. Training sessions on the measurement tools were organized quarterly to ensure that the assessments were conducted uniformly to avoid inter- and intra-observer variability. The interviewers underwent frequent revalidation of their interviewing techniques to avoid biases and to ensure the quality of the data.

### Measurement tools

Invitation letters and consent forms were composed in Maltese and English. Back translation from the English version to Maltese version then back to English version was performed. In line with the WHO STEPS framework, a validated questionnaire-based assessment, simple physical measurements (blood pressure, height, weight and waist and hip circumferences) and biochemical measurements (FBG, lipid profile and glucose tolerance, as measured using an OGTT for the selected participants) were carried out [21]. The questionnaire used was a composite of a number of validated tools, details of which are available in the online Supplementary File. The validated measurement equipment was calibrated in accordance with WHO regulations, details of which can also be found in the online Supplementary File [22].

### Age of participants

The study aimed to assess the adult population in Malta. For this reason and also to avoid the requirement for parental consent, the lower age limit was set at 18. Even though both pre-diabetes and T2DM are being diagnosed at increasingly early ages in many countries, the prevalence in those under 18 would still be relatively low compared with that in the older population [23]. The upper age limit was set at 70 years as beyond this age, the presence of long-standing diabetes or incident disease is more common [24]. Approximately 20% of those older than 65 have diabetes and studying older age groups may skew the true prevalence of diabetes [25]. Research on diabetes in those aged over 70 has shown that glucose diagnoses and glucose control showed no significant cardiovascular disease reduction and some studies even showed excessive deaths in those are undergoing intensive glucose control [24].

### Health examinations

The randomly selected participants were each asked to attend an appointment at various locations in Malta, having fasted for at least 9 h and having abstained from cigarette smoking and physical activity for at least an hour. On registering, each participant was issued with a unique code. An interviewer explained the consent form and allowed time for the participant to read it and ask questions. If the subject agreed to participate, the questionnaire was filled in by the interviewer in accordance with the participant's responses.

While still sitting down, three consecutive blood pressure measurements were taken, and the third blood pressure measurement was recorded (to reduce the impact of white coat hypertension). This was followed by measurements of weight and height using validated weighing scales with a built-in height rod. The participants removed their shoes and excess clothing and emptied their pockets. The waist and hip circumferences were measured using a measuring tape. The waist circumference was measured midway between the lower rib margin and the iliac crest and the hip circumference was measured at the tip of the iliac crest.

Sets of three venous blood samples were collected from each participant for measuring FBG levels, for assessing lipid profiles, and for carrying out a genotyping analysis. The blood samples were collected by skilled phlebotomists, using butterfly needles and a Vacutainer^®^ system. Each blood sample was labelled with the participant's code and put on ice in a cooler bag. Great care was taken with the samples to avoid haemolysis or glycolysis. This involved using preservative tubes and delivering them to the laboratory within 2 h of collection. This was feasible as Malta is a very small country and the laboratory is in a central location, so travelling distances were short.

Each participant was given a copy of all the results of the measurements taken during the examination and they were advised that they would receive a copy of their blood test results by mail within a week and that their results should be discussed with their general practitioner. They were informed that, should their FBG level be 5.6–6.9 mmol/L, this was considered to be a borderline result and so an OGTT would be offered at a later date.

### Blood tests

The assessments of FBG levels and lipid profiles were performed at the Biochemistry Laboratory of the Mater Dei Hospital. Automated and regularly quality controlled COBAS INTEGRA^®^ Analysers machines were used to carry out the tests.

The fasting blood samples to be used to measure FBG levels were collected in fluoride-containing tubes in order to minimize the levels of glycolysis. The FBG levels were measured using hexokinase and glucose oxidase enzyme reactions.

A serum clot activator tube was used to collect blood samples for the lipid profile assessment. This involved assessing each participant's total serum cholesterol, high-density lipoprotein (HDL), low-density lipoprotein (LDL) and triglycerides. These assessments were carried out in light of the link between lipid parameters and diabetes, including the association between the diagnostic criteria for metabolic syndrome (which involves multiple lipid parameters) and the development of T2DM [26]. At the time of formulating this protocol, there was no clear consensus in the literature as to the appropriate fasting period prior to lipid profile testing [27, 28]. Therefore, a 9-h fasting period was chosen in line with the preliminary recommendations in the literature [29].

Using aliquots from each serum sample, the lipid variables were measured as follows: HDL was measured using the clearance method, triglycerides were measured using an enzymatic colorimetric analysis with glycerol phosphate oxidase and 4-aminophenazone total cholesterol was measured using a cholesterol oxidase enzymatic reaction and LDL was measured using the Friedewald formula.

A third venous blood sample was taken in an EDTA tube and stored at −20 °C for later use in a genotyping analysis. This would help us identify any links between different genetic mutations and diabetes mellitus on a population level.

Those participants who were invited to undergo the OGTT were advised to follow their regular diet before the test but to fast the night and the morning before the test. An initial FBG sample was taken from each participant in a fluoride-containing tube. The participants were then given 75 g of glucose and additional blood glucose samples were collected after 1 and 2 h.

### Data input and analysis

A single fieldworker inputted the data in order to avoid bias. The data inputting was performed using a secure online software program that matched the format of the survey questionnaire and also allowed each participant's measurements to be added. The software was programmed to carry out data validation (which included setting upper and lower limits for the various variables such as age) to ensure the quality of the data. The data were then transferred to a spreadsheet program for analysis. The analysis was carried out using IBM SPSS version 21 for Mac.

The participants' questionnaires were cross-referenced with the online versions to check the accuracy of the online data. This process involved cross-referencing several randomly selected questionnaires with the online version at the end of each month.

## Results

A number of lessons were learnt while conducting the survey, as shown in Table 2. A pilot test was conducted to identify each fieldworker's strengths and ensure that the fieldworkers focused on the tasks that they excelled in during the actual survey. Another lesson learnt was that the waist and hip circumferences should be measured after the height and weight measurements. This was because once the participants had removed all their excess clothes and other items, it was easier to continue with the circumference measurements and it helped to prevent inter-observer variability. Care was taken to take each participant's waist circumference measurement as the participant breathed out.

**Table 2.** Summary of the lessons learnt from the survey

Early appointments were appreciated by the participants aged 50 and over
Holding health examinations in each town centre led to good response rates
Flexible appointment dates and times led to good the response rates
Measuring the waist and hip circumference just after measuring weight and height was found most appropriate and acceptable by participants
Carrying out an interview, followed by health examination measurements and then the collection of blood was acceptable to the participants
Explanations of the whole process prior to and during the survey made the experience more enjoyable for the participants
Explanations of the participants' measurements at the end of their health examinations were appreciated by the participants

A health examination hub (where the interviews, collection of blood samples and measurements took place) was set up every weekend at different local government health clinics situated throughout the Maltese Islands. Holding the health examinations in each town health clinic was an effective approach as the participants found it easier to attend when they did not need to travel large distances. The randomly selected participants from each town received a postal invitation letter 2 weeks prior to their appointment, together with an explanation of the aim of the study, the benefits of attending, and an appointment date and time. Participants were given the choice to change their appointment date and time to a more suitable alternative (including a weekday appointment) and to attend a clinic in a different town. This flexibility encouraged those who wanted to attend to participate. The early appointments (7.00 am–7.30 am) were found to be favoured by those over 50, while the later appointments (8.30 am–9.00 am) were favoured by the younger population. The last appointment was set at 9.30 am for two reasons: participants needed to be fasted so late appointments would not be well tolerated and the blood samples needed to be taken to the laboratory within 2 h after they were collected.

The interviews were challenging despite the fact that the interviewers were well trained because there was a risk of information bias as some of the answers depended on the participants' knowledge and memory skills.

At the end of the health examinations, participants often expressed their gratitude for the experience, for being kept informed of their measurement results throughout the processes (where possible) and for being given a copy of their measurement results.

## Discussion

Regular cross-sectional prevalence studies of non-communicable diseases can provide an evidence base to aid public health professionals (including policy makers) in developing strategies to prevent and manage the diseases. Prevalence studies can be technically challenging, laborious and expensive, so they are rarely conducted. Our protocol used a multidisciplinary approach and was based on up-to-date findings from the literature in order to collect cross-sectional epidemiological data at minimal costs and with a minimal number of fieldworkers. The evaluation of the study showed that the protocol set out an effective method for obtaining high-quality data. Although the protocol focused on how to conduct a T2DM prevalence study and to collect data on the determinants of this disease, the protocol can be modified to accommodate the collection of data on other non-communicable diseases.

### Study limitations

Although great care was taken to reduce bias, the study involved data collected from people who may have provided inaccurate data and may not have fasted as advised. Also, participants who had normal FBG levels but abnormal post-prandial levels (indicative of pre-diabetes or diabetes) could have been missed by the study.

## Conclusion

This protocol sets out the methods used in a prevalence study in Malta, which provided essential data that will be used to develop an up-to-date national policy on diabetes (which will include the development of prevention strategies). The study utilized validated measurement tools and was generally found to be acceptable by the participants. The study provided data on the national prevalence of diabetes, IFG, IGT, obesity, regular physical activity, alcohol consumption and tobacco consumption, together with information on the important associations between the potential risk factors and diabetes, and, for the first time, information on the genetics associated with the development of diabetes.

## Supplementary material

The supplementary material for this article can be found at https://doi.org/10.1017/gheg.2016.18.
